# Zbtb34 promotes embryonic stem cell proliferation by elongating telomere length

**DOI:** 10.18632/aging.204285

**Published:** 2022-09-12

**Authors:** Zheng Liu, Xinran Wei, Yue Gao, Xiaodie Gao, Xia Li, Yujuan Zhong, Xiujuan Wang, Chong Liu, Tianle Shi, Jiabin Lv, Tao Liu

**Affiliations:** 1 College of Medical Laboratory Science, Guilin Medical University, Guilin, Guangxi 541004, China; 2 Clinical Laboratory, Hospital Affiliated to Guilin Medical University, Guilin, Guangxi 541001, China; 3 Guihang Guiyang Hospital Affiliated to Zunyi Medical University, Guiyang, Guizhou 550027, China

**Keywords:** Zbtb34, zinc finger, telomere, Pot1b, mouse embryonic stem cells

## Abstract

Zbtb34 is a novel zinc finger protein, which is revealed by biological software analysis to have 3 zinc fingers, but its functions remain unknown. In this study, mouse *Zbtb34* cDNA was amplified by PCR and inserted into the plasmid pEGFP-N1 to generate Zbtb34-EGFP fusion protein. The upregulation of Zbtb34 in mouse embryonic stem cells promoted telomere elongation and increased cell proliferation. In order to understand the above phenomena, the telomere co-immunoprecipitation technique was employed to investigate the relationship between Zbtb34 and telomeres. The results indicated that Zbtb34 could bind to the DNA sequences of the telomeres. Alanine substitution of the third zinc finger abolished such binding. Since Pot1 is the only protein binding to the single-stranded DNA at the end of the telomeres, we further investigated the relationship between Zbtb34 and Pot1. The results revealed that the upregulation of Zbtb34 decreased the binding of Pot1b to the telomeres. Through the upregulation of Pot1b, the binding of Zbtb34 to the telomeres was also reduced. In conclusion, we showed that the main biological function of Zbtb34 was to bind telomere DNA via its third ZnF, competing with Pot1b for the binding sites, resulting in telomere elongation and cell proliferation.

## INTRODUCTION

ZnF (zinc finger) proteins are the most abundant proteins in eukaryotic genomes [1], which are categorized into several types, namely C2H2, C2HC, C2HC5, C2C2, CCCH, C3HC4, C4, C4HC3, C6 and C8 based on the position as well as the number of histidine and cysteine residues [2, 3]. The functions of ZnF proteins are extraordinarily diverse, which have roles in transcriptional activation, protein degradation, DNA repair, cell migration and cell apoptosis [4, 5]. Most ZnF proteins bind to cognate DNA in the nuclei to regulate the expression of target genes [6]. In addition to DNA, many ZnF proteins also bind to proteins or RNA, which fold into a mini domain around a central zinc ion and ‘grab’ the DNA/RNA through the surrounding ZnFs [7].

Zbtb34 (zinc finger and BTB domain containing 34) is a novel zinc finger protein that was first reported by Qi et al. in 2006 [8], who found that Zbtb34 was expressed ubiquitously in most adult human tissues and was mainly localized to the nuclei. Over the next ten years, there had been few studies on Zbtb34. It was found in a previous study that reactive oxygen species-induced anticancer agents could kill cancer cells by downregulating the miR-27a or miR-17/miR-20a [9], which could regulate the expression of Zbtb34, resulting in the suppression of the expression of specificity protein (Sp) transcription factors [10]. This result indicates that Zbtb34 is a transcriptional repressor. It was found in a recent study that Zbtb34 and lnc-CPLC strongly correlated in colorectal cancer progression, indicating that lnc-CPLC/miR-4319/Zbtb34 signal axis played a role in regulating the development of colorectal cancer [11].

Mouse embryonic stem cells (mESCs) are considered to be a unique tool for many researchers since they have the ability to self-renew and differentiate into all cell types [12]. mESCs derived from knock-in/knockout blastocysts are very useful for studying mutant mice, especially when the phenotype is embryonic-lethal at early stages of development [13]. In the present study, we found that Zbtb34 promoted the proliferation of embryonic stem cells and elongated telomere length by competing with Pot1 (protection of telomere 1) b for the telomere binding sites. Since the deletion of both Pot1a and Pot1b resulted in early embryonic lethality [14], the biological functions of Zbtb34 are investigated through various experiments on mESCs.

## RESULTS

### Zbtb34 induces the proliferation of mESCs

**Figure 1:**
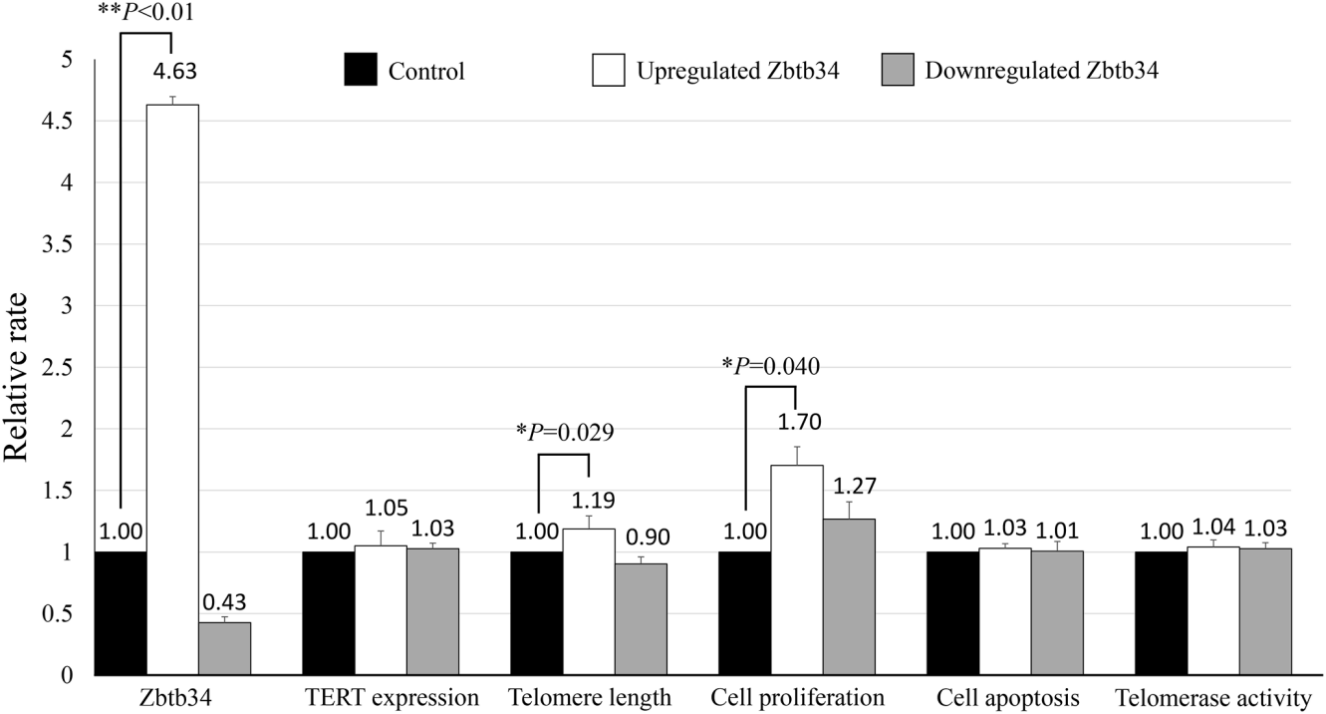
**Zbtb34 elongates telomere length and increases cell proliferation.** The mESCs were transfected with a Zbtb34-EGFP vector or Zbtb34-siRNA. The values of Zbtb34, TERT expression, telomere length, cell proliferation, cell apoptosis and telomerase activity are expressed as fold changes compared with control values normalized to 1. ^*^P < 0.05, ^**^P < 0.01. Abbreviations: mESCs: mouse embryonic stem cells; TERT: telomerase reverse transcriptase.

MTT assay demonstrated that the upregulation of Zbtb34 induced the proliferation of mESCs (Figure 1). However, the rate of apoptotic cells was not significantly different between the upregulated- and downregulated-Zbtb34 mESCs through a flow cytometry analysis.

### Zbtb34 elongates telomere length

The telomere length was detected in mESC lines transfected with Zbtb34-EGFP or siZbtb34. The result revealed that the upregulation of Zbtb34 significantly elongated the telomere length (Figure 1), which, interestingly, did not affect the expression of TERT (telomerase reverse transcriptase), indicating that Zbtb34 did not regulate the TERT expression. Moreover, there was no difference in telomerase activity between upregulated- and downregulated-Zbtb34 mESCs (Figure 1).

### Zbtb34 has 3 zinc finger motifs

**Figure 2:**
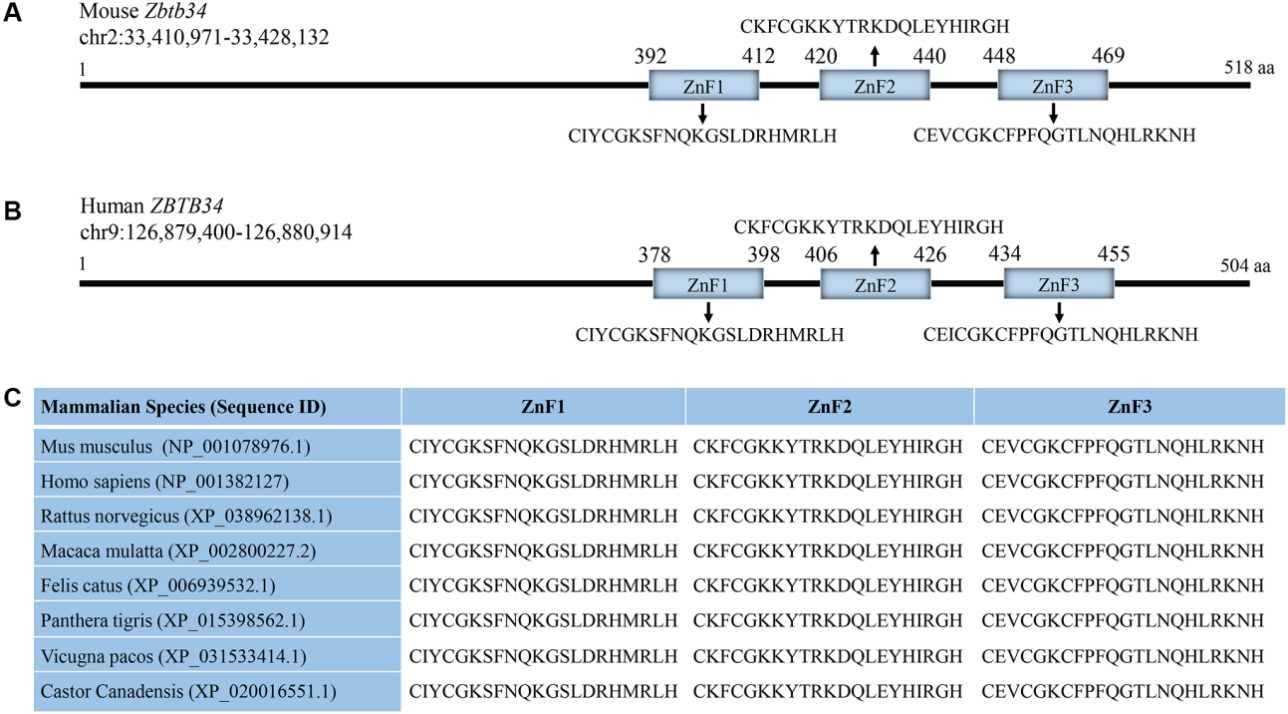
**Zbtb34 contains 3 highly-conserved ZnFs.** (**A**) Mouse *Zbtb34* is located on Chromosome 2. (**B**) Human *Zbtb34* is located on Chromosome 9. (**C**) The alignment of the amino acid sequences within ZnFs is highly conserved among different mammalian species. The number of parentheses is the accession number. Abbreviation: ZnF: zinc finger.

Mouse Zbtb34 was predicted to have 3 C2H2 ZnFs (Figure 2A). Human Zbtb34 also contains 3 ZnFs, but its location is different from that of the mouse (Figure 2B). Since our research group is working on mESCs, in this study, we aim to clarify the functions of mouse Zbtb34. The alignment of the amino acid sequences of *Mus musculus*, *Homo sapiens*, *Rattus norvegicus*, *Macaca mulatta*, *Felis catus*, *Panthera tigris*, *Vicugna pacos* and *Castor canadensis* shows that the amino acids within ZnFs are highly conserved among different species (Figure 2C).

### The ZnF3 is necessary for Zbtb34 binding to telomeres

**Figure 3:**
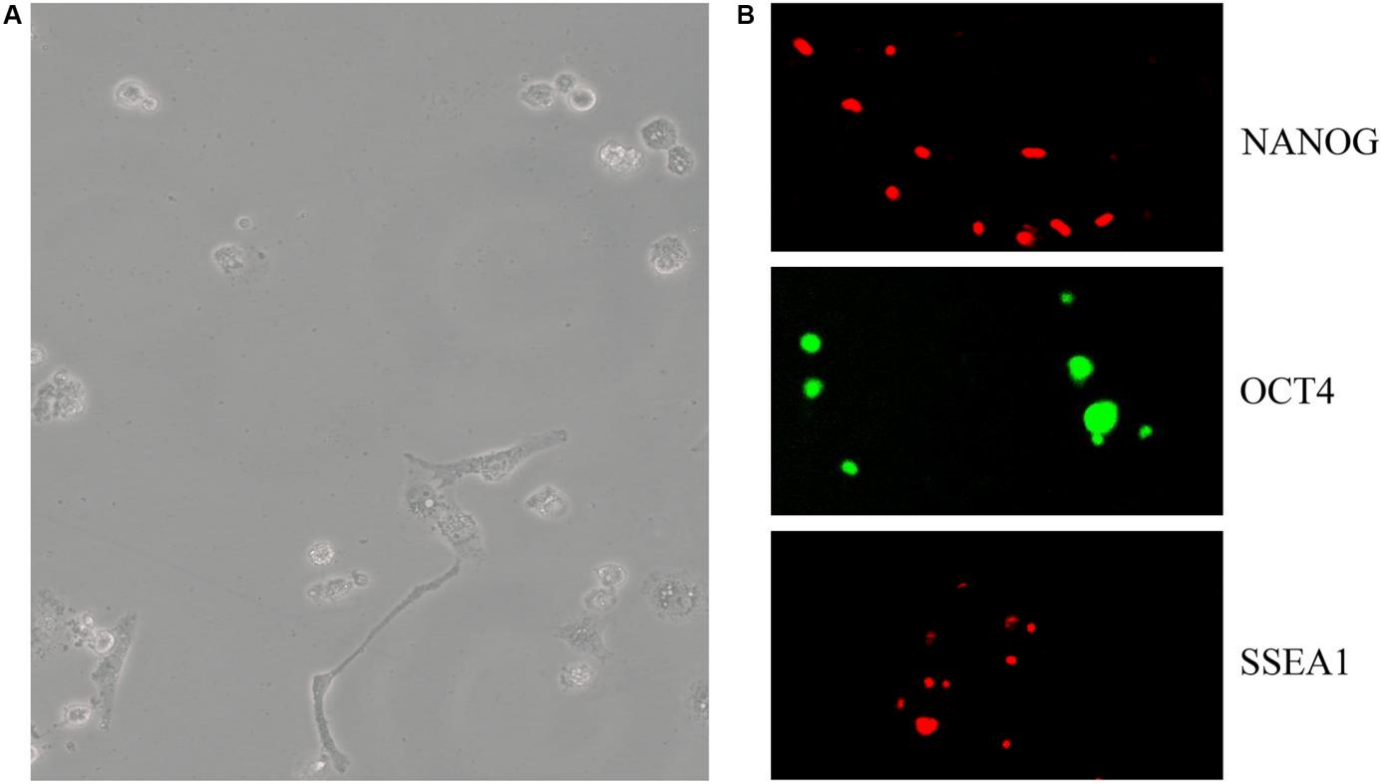
**mESCs express pluripotency markers.** (**A**) C57BL/6 mESC culture in OriCell C57BL/6 × 129 medium. (**B**) The pluripotency marker NANOG (red), OCT4 (green), SSEA1 (red) and staining was visualized through fluorescent microscopy. Abbreviation: mESCs: mouse embryonic stem cells.

**Figure 4:**
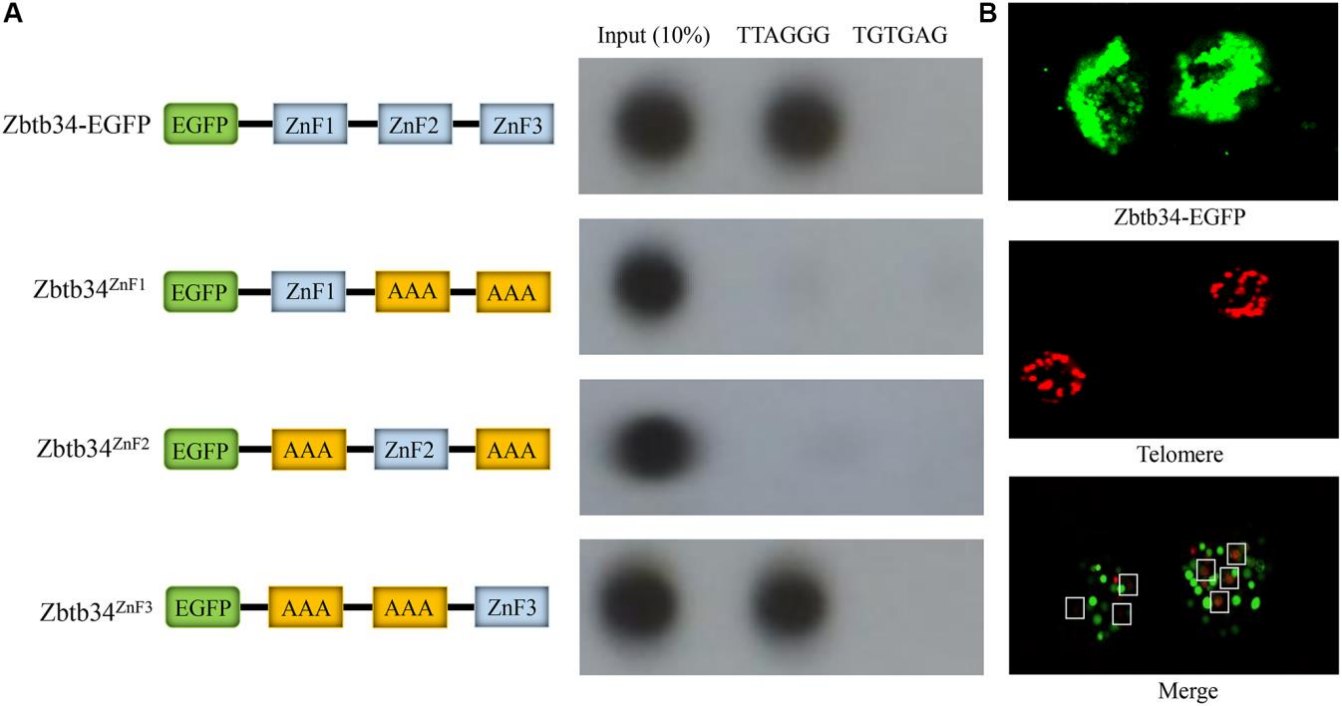
**Zbtb34 binds to the telomere DNA.** (**A**) The ZnFs within Zbtb34 of mESCs are analyzed through telomere co-immunoprecipitation. Schemata represents the construct of the Zbtb34-EGFP vector and the alanine substitution of ZnF1, ZnF2 or ZnF3. The results show that Zbtb34 is co-immunoprecipitated with the sequence (TTAGGG) of the telomeres. The association of ZnF1, ZnF2 or ZnF3 with telomere DNA is further analyzed. The results reveal that ZnF3 is co-immunoprecipitated with the telomere DNA. The random sequence (TGTGAG) is the control. (**B**) Fluorescence *in situ* hybridization shows the localization of Zbtb34 (green) and telomeres (red). The dots in the white boxes indicate the merger of green and red dots. Abbreviations: ZnF: zinc finger; mESCs: mouse embryonic stem cells.

C57BL/6 mESCs cultured in OriCell C57BL/6 × 129 medium (Figure 3A) were positive for the pluripotency markers NANOG (red), OCT4 (green), and SSEA1 (red) (Figure 3B). Using the telomere ChIP, we found that Zbtb34 could bind to the telomere DNA sequences (repeat TTAGGG) (Figure 4A). The co-location of Zbtb34 and telomeres was confirmed through the FISH (Fluorescence *in situ* hybridization) experiment (Figure 4B). We further investigated the functions of each zinc finger motif. A series of alanine scan mutants were generated to substitute the amino acids within each ZnF. The absence of ZnF2/ZnF3 and ZnF1/ZnF3 abolishes the Zbtb34 binding to telomeres (Figure 4A). However, the absence of ZnF1/ZnF2 (Figure 4A) did not affect the binding between Zbtb34 and telomeres, which suggests that ZnF3 at the C-terminus of amino acid protein 448 to 469 is necessary for Zbtb34 binding to the telomeres.

### Zbtb34 competes for the binding sites with Pot1b

**Figure 5:**
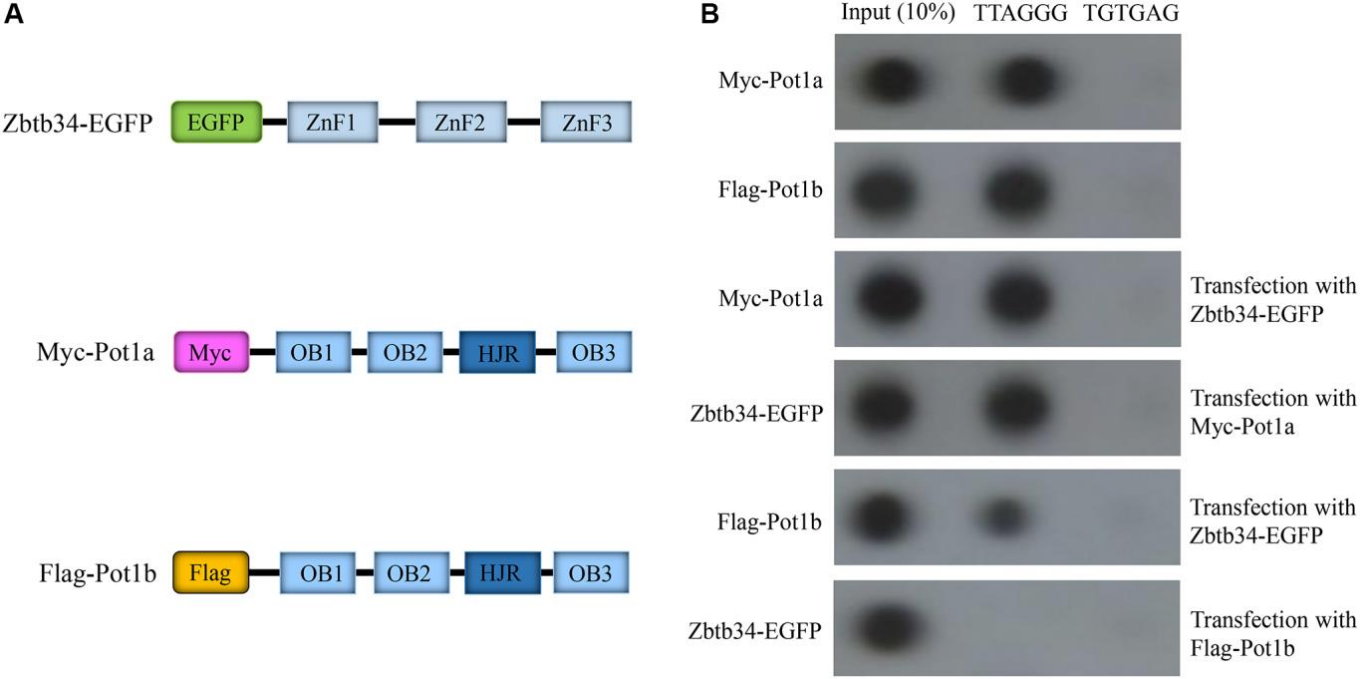
**Zbtb34 competes for the binding sites on telomeres with Pot1b.** (**A**) Schemata represents the Myc-Pot1a and Flag-Pot1b constructs. (**B**) Telomere co-immunoprecipitation shows the association of Zbtb34 and Pot1a or Pot1b with telomere DNA in mESCs. The random sequence (TGTGAG) is the control. Abbreviations: ZnF: zinc finger; OB: oligonucleotide binding; HJR: holiday junction resolvase; mESCs: mouse embryonic stem cells.

Since Pot1 was the only protein binding to the single-stranded DNA TTAGGG at the end of the telomeres, we investigated the relationship between Zbtb34 and Pot1a or Pot1b, two highly homologous isoforms in mice. The results revealed that the upregulation of Zbtb34 decreased the binding of Pot1b to telomeres, which also reduced the binding of Zbtb34 to the telomere DNA (Figure 5). This result indicates that Zbtb34 and Pot1b have the same binding site at the end of the telomeres and a competitive relationship in telomere binding. Interestingly, the upregulation of Zbtb34 does not affect the binding between Pot1a and telomeres.

## DISCUSSION

To date, the relationship between ZnF proteins and telomeres remains largely unknown [15]. Our study demonstrated that Zbtb34 could bind to telomere DNA directly. Since the alanine substitution of ZnF3 inhibited the binding, it suggested that the presence of ZnF3 was necessary for Zbtb34 to bind to telomere DNA. Based on its localization at the telomeres, we reasoned that Zbtb34 might play a role in telomere length regulation. The result confirmed our hypothesis that the upregulation of Zbtb34 promoted telomere elongation. In recent years, the association between ZnF proteins and telomeres has been pointed toward in more and more studies. The most prominent example is the ZBTB48, which can bind to long telomeres through its ZnF11, competing with the telomeric-repeat binding factors TRF1 and TRF2, setting an upper limit for telomere length through triggering telomere trimming [16]. ZBTB10 is another reported protein that can bind to telomeres, which binds to the telomeres with its two C2H2 zinc fingers and interacts with the N-terminal domain of TRF2 [17]. However, ZBTB10 is not involved in telomere homeostasis, indicating that the functions of ZBTB10 binding to telomeres need to be further studied. An interesting finding in this study is that only the third ZnF of Zbtb34 binds to the telomeres, while the functions of the other two ZnFs are unclear. It was found in previous studies that ZBTB48 and ZBTB10 were involved in the progression of different cancers [18, 19]. Zbtb34 has also been shown to play a role in tumor growth as a transcriptional repressor [20]. Therefore, we believe that the other two ZnFs in Zbtb34 are involved in transcriptional repression of tumor growth.

**Figure 6:**
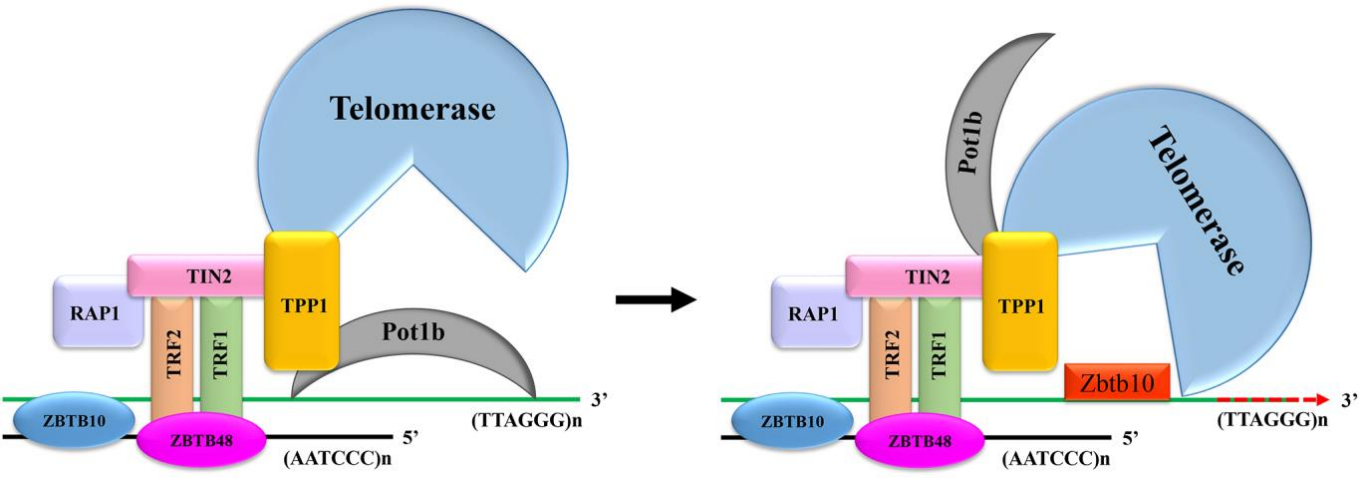
**The mechanism for Zbtb34 to regulate telomere length.** When the expression of Zbtb34 is more than that of Pot1b, Zbtb34 prevents Pot1b from binding to telomere DNA, which increases the opportunity for telomerase to access telomere DNA. The telomerase adds DNA repeats (TTAGGG)n to the 3′-ends of telomeres. When the expression of Pot1b is more than that of Zbtb34, Pot1b inhibits telomerase recruitment to telomeres, resulting in telomere shortening with cell division.

Telomeres consist of DNA repeating sequences (TTAGGG) and shelterin complex at the end of chromosomes, protecting the chromosomes from fusion or degradation [21, 22]. This shelterin complex consists of six telomere-specific proteins, namely TRF1, TRF2, TIN2, POT1, TPP1 and RAP1 (Figure 6) [23]. TIN2 plays a pivotal role in suppressing ATM and ATR signaling pathways through interacting with TPP1/POT1 heterodimer as well as in stabilizing TRF1 and TRF2 at the telomeric DNA [24]. TRF2/RAP1 heterodimer prevent telomere fragility [25]. Among these shelterin proteins, only Pot1 directly recognizes single TTAGGG repeats at the 3′ telomere terminus [26]. Mammalian shelterin proteins Pot1 and TPP1 form a stable heterodimer regulating telomerase-mediated telomere extension [27]. Pot1 plays a negative role in preventing telomerase recruitment to telomeres, resulting in telomere shortening [28]. In mice, Pot1 has two highly homologous isoforms, namely Pot1a and Pot1b. However, the functions of these two isoforms are ill-defined because of the lack of inhibitors that can distinguish them [29]. Since Pot1 can bind to single TTAGGG repeats, we speculated that Pot1 and Zbtb34 had the same binding site on the telomeres. To test this hypothesis, we generated constructs Myc-Pot1a and Flag-Pot1b. The upregulation of Zbtb34 in mESCs caused a drastic reduction in the localization of Pot1b at the telomeres. Similarly, the upregulation of Pot1b also affected the localization of Zbtb34 at telomeres, suggesting that Zbtb34 had a competitive relationship with Pot1b in telomere binding. It can be concluded from our experimental results that Zbtb34, ZBTB48 and ZBTB10, which are 3 ZnF proteins known to bind to telomeres, have different binding activity.

The depletion of Pot1 has been demonstrated to expose the 3′ telomere terminus and significantly promote telomere elongation [30, 31], which regulates telomere length by leaving telomeres occasionally, allowing telomerase to bind to telomeres, thus resulting in telomerase-mediated telomere lengthening [32]. Therefore, we speculated that the mechanism for Zbtb34 to facilitate telomere elongation was that Zbtb34 physically occupied the position of Pot1b at the telomeres and then helped telomerase interact with 3′ telomere terminus, resulting in telomere elongation (Figure 6). This result is consistent with the study of Gu et al., who recently found that Pot1 negatively regulated telomere length by inhibiting telomerase recruitment to telomeres [33]. Pot1b has been found to have important protective functions for telomeres. Pot1b knockout cells and mice resulted in telomere degradation caused by initiating a DNA damage response [34]. Based on the above experimental results, we speculated that Zbtb34 occupied the telomere binding sites, preventing the activation of DNA damage response. It also provided space for telomerase to bind to telomere DNA, resulting in telomere elongation and mESC proliferation (Figure 6). Telomere length maintenance is important to facilitating cell division [35], which is also critical for the unlimited self-renewal, pluripotency and chromosomal stability of mESCs [36]. The risk of diseases associated with a reduced cell proliferation, including aging or aging-associated diseases, is correlated with an increase in the shortening of telomeres [37]. An interesting finding of this study was that Zbtb34 did not affect the localization of Pot1a at telomeres, indicating that Pot1a and Pot1b played different roles in maintaining telomere functions in mESCs. The biological functions of Pot1a and Pot1b in telomere homeostasis need to be further investigated.

## CONCLUSIONS

In conclusion, our results demonstrated that Zbtb34 could bind to telomeres, and for which ZnF3 was responsible. Furthermore, the upregulation of Zbtb34 reduced the Pot1b binding to telomere DNA sequences, indicating that the main function of Zbtb34 was to compete for the binding sites on telomeres through Pot1b, resulting in the telomere elongation and cell proliferation. The limitation of this study is that the functions of the other two ZnFs are still unclear. Therefore, ZnF1 and ZnF2 in Zbtb34 need to be further investigated.

## MATERIALS AND METHODS

### Constructs

*Zbtb34* cDNA was amplified by PCR using *Pfu* Turbo DNA polymerase (Agilent Corporation, USA) and appropriate primers (Table 1). After digesting the PCR products with *Xho I* and *BamH I*, the cDNA fragments were inserted into the *Xho I*/*BamH I* site of pEGFP-N1 to generate Zbtb34-EGFP fusion protein. Mouse Pot1 has two highly homologous isoforms, namely Pot1a and Pot1b, whose cDNA was amplified by PCR and then inserted into the *Xho I*/*EcoR I* sites of eukaryotic-expression vector pcDNA3.1 to generate pcDNA3.1-Myc-Pot1a as well as pcDNA3.1-Flag-Pot1b. The primers used in the study are listed in Table 1.

**Table 1:** The primers and siRNA used in this study.

**Primers**	**Sequences (5′→3′)**	**Target**
*Zbtb34* cDNA	Forward: CTCGAG ATGGAAAGCACCCTGG	Zbtb34
	Reverse: GAGCTCTTAGTCAGGCGCATC	
*Pot1a* cDNA	Forward: CTCGAGATGTCTTTGGTTTCAAC	Pot1a
	Reverse: GAATTCCTAGACAACATTTTCTG	
*Pot1b* cDNA	Forward: CTCGAGATGTCTTCGGCCCCAG	Pot1b
	Reverse: GAATTCCTAGATGTGTCTTC	
ZnF1	Forward: GTTGATCGCTGCTGCTGCTGCTGCTGCTGCTGCTGCTGCTGCTGCTGCTGCTGCTGCTGCTGCTGCTATGGG	ZnF1
	Reverse: AGCAGCAGCAGCAGCAGCAGCAGCAGCAGCAGCAGCAGCAGCAGCAGCAGCAGCAGCAGCAGCGATCAACCTCTCG	ZnF1
ZnF2	Forward: TTTGTGGCTGCTGCTGCTGCTGCTGCTGCTGCTGCTGCTGCTGCTGCTGCTGCTGCTGCTGCTGCTGCTACTGATG	ZnF2
	Reverse: AGCAGCAGCAGCAGCAGCAGCAGCAGCAGCAGCAGCAGCAGCAGCAGCAGCAGCAGCAGCAGCCACAAAGGGGGTGATC	ZnF2
ZnF3	Forward: ATTCCGAGCTGCTGCTGCTGCTGCTGCTGCTGCTGCTGCTGCTGCTGCTGCTGCTGCTGCTGCTGCTGCTGCTCCGGGGGTCAC	ZnF3
	Reverse: AGCAGCAGCAGCAGCAGCAGCAGCAGCAGCAGCAGCAGCAGCAGCAGCAGCAGCAGCAGCAGCAGCTCGGAATGGTTTGTC	ZnF3
siRNA 1	Guide: UUCACUAAGCUAAAUUCAGCA	Zbtb34
	Passenger: CUGAAUUUAGCUUAGUGAAGG	Zbtb34
siRNA 2	Guide: AACAGAAAAUAACCAUUAGAA	Zbtb34
	Passenger: CUAAUGGUUAUUUUCUGUUGA	Zbtb34

### Bioinformatics analysis

Using the batch PWM predictor software (http://zf.princeton.edu/index.php), the positions of ZnFs within Zbtb34 were predicted. The Zbtb34-siRNA was designed through siDirect version 2.0 (http://sidirect2.rnai.jp/).

### Alanine scanning mutagenesis

The expression vectors Zbtb34^ZnF1^ (alanine substitution of ZnF2 and ZnF3), Zbtb34^ZnF2^ (alanine substitution of ZnF1 and ZnF3) and Zbtb34^ZnF3^ (alanine substitution of ZnF1 and ZnF2) were constructed using site-directed mutagenesis kits (Sangon Biotech, Shanghai, China). Details of primers used in the study are listed in Table 1. The sequence and the mutants were confirmed through DNA sequencing.

### Cell culture and transfection

C57BL/6 mESCs generated from the inner cell mass of a 3.5-day mouse blastocyst and OriCell C57BL/6129 culture media comprising fetal bovine serum, glutamine as well as 2-mercaptoethanol were purchased from Cyagen Bioscience Inc, Suzhou, China. The cells were transfected with constructs (4.0 μg) using Liperfactamine 3000 (Invitrogen, USA) and stained with Hoechst 33342 (10 mg/ml). The fluorescence was visualized through an Olympus BX51 fluorescence microscope.

### Immunocytochemistry

Immunocytochemistry assay was performed as previously described [38]. The cells were fixed in 4% paraformaldehyde solution at room temperature for 30 min, and then washed and permeabilized in 0.5 ml of PBST solution for 30 min. Cells were then treated with primary antibodies Rabbit monoclonal to OCT4 (Abcam, ab200834), Rabbit monoclonal to CD15/SSEA1, (Abcam, ab135377), Rabbit monoclonal to NANONG (Abcam, ab203919), overnight at 4°C followed by fluorochrome labelled secondary antibodies Goat anti-Rabbit IgG Alexa Fluor 488 (Abcam, ab150077) and Goat Anti-Rabbit IgG Alexa Fluor 647 (Abcam, ab150083) at room temperature for 1 h. The images were captured with an Olympus BX51 microscope.

### Fluorescence *in situ* hybridization (FISH)

mESCs transferred through vectors grown on coverslips were fixed for 5 min in 1% paraformaldehyde. Cells were then washed in PBS for 3 times. A Cy5-[CCCTAA]_3_-labelled probe (Sangon Biotech, Shanghai, China) was added to the coverslips in a buffer containing 70% formamide, 1 mg/ml blocking solution (1 mg/ml BSA, 3% goat serum, 0.1% Triton X-100, 1 mM EDTA in PBS) and 10 mM Tris-HCl pH 7.2, denatured at 80°C for 5 min, and hybridized for 1 h at room temperature in the dark. The coverslips were washed twice with 70% formamide and 10 mM Tris-HCl with a pH of 7.2 for 15 min each, which were then mounted for image analysis. This experiment was repeated for 3 times independently.

### Telomere chromatin immunoprecipitation (ChIP)

The method refers to the study of Liu [39]. mESCs were cross-linked in 1% formaldehyde for 60 min and then added to 1 ml of SDS lysis buffer (1% SDS, 10 mM EDTA, 50 mM tris- HCl and 1 mM PMSF). Ultrasonically-induced cell lysis was used to obtain chromatin fragments <1 kB. The supernatant was added to 1 ml of IP buffer (0.01% SDS, 1.1% Triton X-100, 1.2 mM EDTA, 16.7 mM Tris, 167 mM NaCl and 1 mM PMSF), and then incubated with antibodies at 4°C overnight. Eluting buffer (10 mM Tris, 5 mM EDTA, 1% Triton X-100 and 0.1% SDS) was added at 65°C for 30 min and then de-crosslinked at 65°C. The protease K with a final concentration of 100 μg/ml was added and incubated at 50°C for 2 h. The immunoprecipitated DNA was purified through QIAEX II System. DNA was denatured and hybridized to a GeneScreen plus hybrid membrane, which was hybridized with a ^32^P-labelled-telomere repeats (4× TTAGGG) probe overnight at 42°C. The exposure and scanning were performed with a typhoon 9500 imager (GE Healthcare Life Sciences, USA). The anti-GFP (No. D191040), anti-Myc (No. D110006) and anti-Flag (No. D110005) antibodies were purchased from Sangon Biotech, Shanghai, China. This experiment was repeated for 3 times independently.

### Real-time PCR

The quantitative real-time PCR was performed to determine the mRNA abundance of telomerase reverse transcriptase (TERT) and Zbtb34. mESCs were transfected through a Zbtb34-EGFP vector or Zbtb34-siRNA for 48 h. Total RNA was isolated from the cells using RNAzol (Sigma-Aldrich, China). mRNA was reversely-transcribed to cDNA using RevertAid first-strand cDNA synthesis kits (No. K1621, Sangon Biotech, Shanghai, China). Real-time PCR was run in a Roche 480 real-time PCR system. Master mix in each reaction tube includes cDNA, ddH_2_O, SYBR Green, forward and reverse primer of genes (Table 1). This experiment was repeated for at least 3 times independently.

### Telomere length measurement

Real-time PCR was employed to measure telomere length using a relative mouse telomere length quantification qPCR assay kit (No.M8908, Shanghai ZhongQiaoXin Zhou Biotechnology, China) according to the manufacturer’s protocol. 200 μL of ddH_2_O was added to the telomeres and the SCR primer set. The 20 μL of qPCR in reaction included 1 ng of genomic DNA template, 2 μL of telomere primers and SCR primers, 10 μL of 2× qPCR master mix and ddH_2_O. The qPCR process lasted for 10 min at 95°C, followed by 32 cycles (denaturation at 95°C for 20 s; annealing at 52°C for 20 s and extension at 72°C for 45 s). This experiment was repeated for at least 3 times independently with similar results.

### Telomerase activity measurement

Telomerase activity was analyzed using a telomerase enzyme-linked immunosorbent assay (ELISA) kit (No. ml037815, Mlbio, China). Briefly, 1 × 10^6^ mESCs were lysed with 200 μL of RIPA lysis buffer (Beyotime, China). Subsequently, the protein supernatant and 50 μL of biotin-labeled antibodies were mixed at 37°C for 1 h. After washing with PBS to remove the unbound antibodies, 80 μL of horseradish peroxidase was added and incubated for 30 min at 37°C. 50 μL of substrates A and B were then added to each well after the washing was completed. Finally, after reading the absorbance values at 450 nm, telomerase activity (IU/L) of cells was calculated based on the standard curve created according to the standards of the kit. This experiment was repeated for 3 times independently with similar results.

### MTT assay

A total of 1 × 10^4^ cells per well were seeded in 96-well plates and grew to 80% confluence. The next day, the culture medium was completely removed, and 90 μL of PBS was added to each well. Then, the plates were incubated for 1 h at room temperature. 50 μL of MTT solution (5 mg/ml) was added, and cells were incubated in a CO_2_ incubator in the dark for 2 h. The medium was removed, and formazan crystals formed by the cells were dissolved in 500 μl of DMSO, which were then transferred to 96-well plates. The absorbance was read at a wavelength of 490 nm through a PT-3502C plate reader (Potenov, Beijing, China). This experiment was repeated for 3 times independently with similar results.

### Flow cytometry

The cells were inoculated into a 6-cm dish and cultured for 48 h until they were adhered to the wall. The cell culture medium was collected in a 10 mL centrifuge tube and washed with PBS after incubation. Subsequently, the cells were centrifuged to remove the supernatant, remixed with binding buffer and mixed with Annexin V-FITC as well as propidium iodide (PI) staining solution. After a thorough mixing, the cells were incubated at room temperature in the dark for 15 min. PBS was added to the cell suspension in the flow tube, and a flow cytometry assay (Beckman Coulter, USA) was performed. This experiment was repeated for 3 times independently with similar results.

### Statistical analysis

The data was expressed by mean ± SD. Differences were considered statistically significant at *P* < 0.05. All statistical analyses were performed using SPSS 20 (SPSS Inc., USA). All experiments were repeated for 3 independent times.
